# Disseminated Sphingomonas paucimobilis Infection Mimicking Tuberculosis in a Patient With Type 3c Diabetes: Case Report

**DOI:** 10.7759/cureus.111989

**Published:** 2026-07-03

**Authors:** Keshao B Nagpure, Sravya Kuchi, Sunita Kumbhalkar, Pallavi Yelne, Ishan Verma, Amol H Dube

**Affiliations:** 1 General Medicine, All India Institute of Medical Sciences, Nagpur, Nagpur, IND; 2 General Medicine Cardiology, All India Institute of Medical Sciences, Nagpur, Nagpur, IND

**Keywords:** cervical lymphadenopathy, disseminated tuberculosis, pulmonary nodules, spingomonas paucimobilis, type 3c diabetes

## Abstract

*Sphingomonas paucimobilis* is a rare opportunistic gram-negative bacillus increasingly reported in immunocompromised individuals. A 45-year-old male presented with right cervical swelling, fever, weight loss, anorexia, jaundice, and a discharging cervical sinus for two weeks. Examination revealed tachycardia, tachypnoea, pallor, icterus, cervical lymphadenopathy with sinus discharge, hepatosplenomegaly, and left-sided chest crepitations. Investigations showed neutrophilic leucocytosis, elevated C-reactive protein (CRP), deranged liver and renal function tests, and uncontrolled diabetes mellitus. Imaging demonstrated necrotic cervical lymphadenopathy, chronic calcific pancreatitis, and multiple bilateral pulmonary nodules with ground-glass opacities. Pus examination was negative for acid-fast bacilli, fungal elements, and a cartridge-based nucleic acid amplification test (CBNAAT) for tuberculosis; however, repeated cultures isolated *Sphingomonas paucimobilis*. Fine-needle aspiration cytology (FNAC) from a pulmonary nodule showed no malignancy and negative CBNAAT. The patient demonstrated marked clinical, laboratory, and radiological improvement with culture-guided piperacillin-tazobactam therapy and optimal glycaemic control. This case underscores the need to consider rare opportunistic infections as important differentials of disseminated tuberculosis.

## Introduction

*Sphingomonas paucimobilis* is an obligate aerobic, non-fermenting Gram-negative bacillus that was originally considered a Pseudomonas until its reclassification in 1977 as a separate genus [1], distributed in soil and water sources, including hospital environments. The bacterium is an opportunistic pathogen predominantly associated with immunocompromised hosts and nosocomial infections, although community-acquired infections have also been increasingly recognized. Despite its traditionally low virulence, recent literature has implicated this organism in a broad spectrum of clinical manifestations, including bacteremia, pneumonia, meningitis, peritonitis, soft tissue infections, and healthcare-associated outbreaks [2]. However, systemic or disseminated involvement remains uncommon and is sparsely described in the literature. In tuberculosis-endemic countries such as India, its pathogenic potential is often overshadowed by more prevalent aetiologies such as tuberculosis and common bacterial pathogens. We report a clinically challenging case of probable systemic involvement associated with *Sphingomonas paucimobilis* infection in a 45-year-old patient with recently diagnosed uncontrolled type 3c diabetes mellitus and chronic calcific pancreatitis, presenting with fever, weight loss, necrotic cervical lymphadenopathy, discharging sinus, pulmonary nodules, and hepatosplenomegaly closely mimicking disseminated tuberculosis.

## Case presentation

A 45-year-old male presented with painless right-sided neck swelling, breathlessness, low-grade fever, weight loss, anorexia, and jaundice for two weeks. One week before hospitalization, the neck swelling ruptured spontaneously with pus discharge. On examination, he had tachycardia (110/min), tachypnoea (24/min), SpO₂ 90% on room air, pallor, and icterus. Multiple matted right cervical lymph nodes (largest 2 × 2 cm), firm, mobile, non-tender, with a sinus discharging serohemorrhagic fluid, were noted in the anterior triangle of the neck (Figure 1).

**Figure 1:**
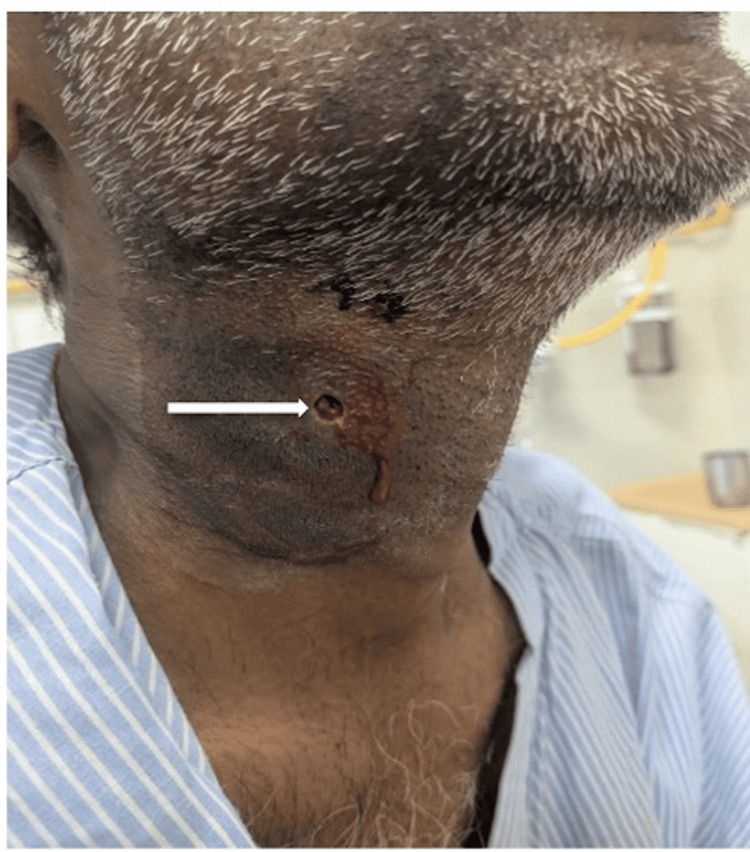
Right cervical lymphadenopathy with discharging cervical sinus (white arrow)

Chest examination revealed crepitations in the left infraclavicular and intrascapular regions. Investigations showed anaemia, neutrophilic leucocytosis, markedly elevated C-reactive protein (CRP), deranged renal and liver function tests with hyperbilirubinemia, and uncontrolled diabetes (Table 1).

**Table 1:** Laboratory investigations on admission and discharge. HbA1c: glycosylated haemoglobin.

Test	Value (On Admission)	Value (on Discharge Day 14)	Normal Range
Hemoglobin	10.4	11	12-15 g/dl
Total leucocyte count	15070	6030	4000-10,000 /mm^3^
Platelet count	1.45	4.04	1.5-4.5 lakh/mm^3^
C-reactive protein	350	1.59	<5 mg/L
Serum creatinine	1.4	0.76	0.6-1.3 mg/dl
Blood urea	98.7	18.6	15 – 39 mg/dl
Bilirubin (Total)	10.54	2.49	0.2 - 1.2 mg/dl
Bilirubin (Direct)	5.43	1.41	0.2 - 0.9 mg/dl
Bilirubin(Indirect)	5.11	1.08	0 - 0.3 mg/dl
Aspartate aminotransferase (AST)	108	25.6	0-45 U/L
Alanine aminotransferase (ALT)	38.2	31.4	0-45 U/L
Serum Albumin	1.98	3.19	3.2-4.5 g/dl
HbA1c	12.9	-	< 5.4 %

Human immunodeficiency virus (HIV), hepatitis B surface antigen (HBsAg), and hepatitis C virus antibody (anti-HCV) were nonreactive. Chest X-ray with upper abdomen showed bilateral heterogeneous opacities suggestive of patchy consolidation & Intrabdominal calcification (Figure 2).

**Figure 2:**
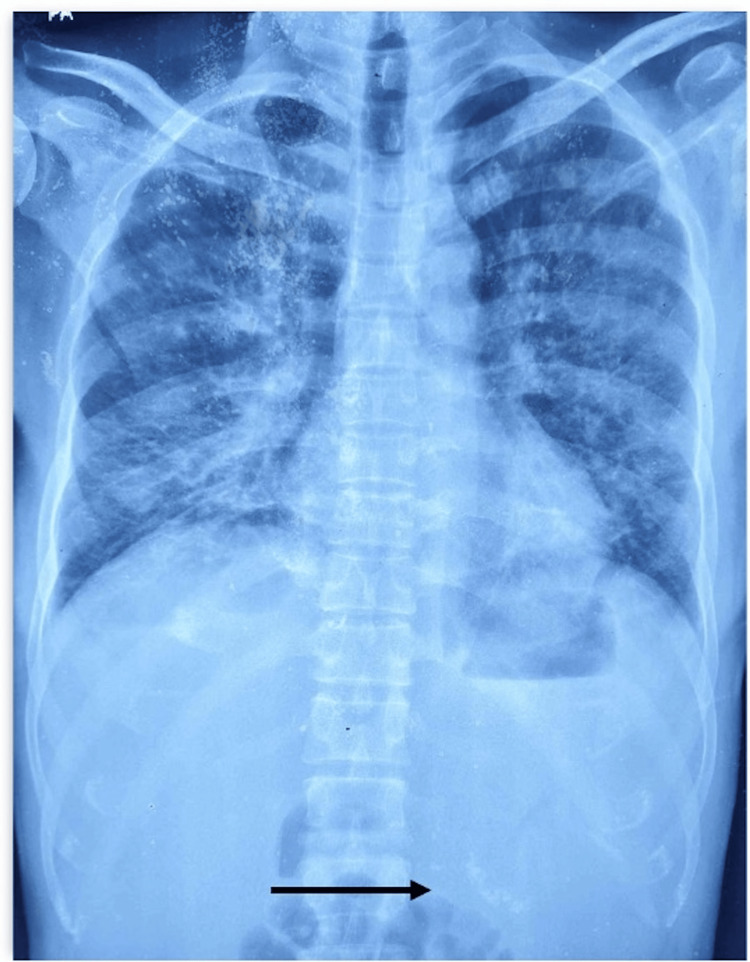
X-ray chest with upper abdomen on admission (day 1). Black arrow shows intra-abdominal calcification.

Ultrasound neck demonstrated multiple necrotic right cervical lymph nodes with a hypoechoic collection extending to the skin surface, forming a sinus tract. Contrast-enhanced computed tomography (CECT) of the thorax and abdomen revealed hepatosplenomegaly with hepatic steatosis, chronic calcific pancreatitis with intraductal and intraparenchymal calcifications (Figures 3A, 3B), and multiple bilateral pulmonary nodules with central necrosis and surrounding ground-glass opacities, a few of which showed spiculated margins (Figures 4A, 4B).

**Figure 3:**
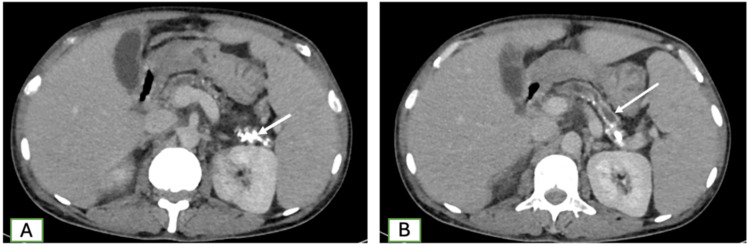
Contrast-enhanced computed tomography abdomen. A&B) showing chronic calcific pancreatitis with intraparenchymal & intraductal calcification (white arrows) with hepatosplenomegaly.

**Figure 4:**
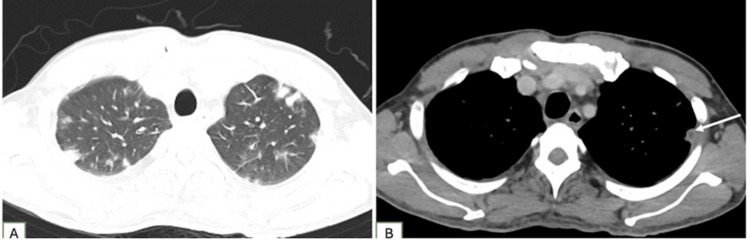
Contrast-enhanced computed tomography (CECT) thorax. A) Multiple variable-sized lung nodules with surrounding ground-glass opacities.  B) Plural-based nodule with central necrosis (white arrow).

Microbiological evaluation of pus from the discharging sinus (two samples) showed abundant pus cells with no organisms on Gram stain, no acid-fast bacilli, negative potassium hydroxide preparation (KOH mount), and negative CBNAAT for Mycobacterium tuberculosis. Culture grew *Sphingomonas paucimobilis*, identified by the automated VITEK system (bioMérieux, Marcy-l'Étoile, France) with susceptibility to piperacillin-tazobactam and ceftazidime, but resistance to carbapenems, fluoroquinolones, and aminoglycosides. Based on clinical, radiological, and microbiological findings, a provisional diagnosis of chronic calcific pancreatitis with type 3c diabetes mellitus, disseminated tuberculosis, and secondary disseminated *Sphingomonas paucimobilis* infection was considered. Antibiotics guided by sensitivity, along with strict glycaemic control, were initiated.

The patient was further evaluated to confirm or exclude disseminated tuberculosis. A CT-guided biopsy of the peripheral lung nodule was planned. However, by the time the procedure was scheduled, repeat imaging performed eight days after initiation of antibiotic therapy demonstrated significant regression of the pulmonary nodules, making procurement of an adequate tissue sample difficult. Consequently, fine-needle aspiration cytology (FNAC) was performed from a small (6 mm) pleural-based necrotic nodule in the left lung. FNAC showed no evidence of malignancy, and CBNAAT was negative for Mycobacterium tuberculosis.

The patient was treated with intravenous piperacillin-tazobactam according to culture and sensitivity results, along with insulin for glycaemic control. The patient demonstrated significant clinical, laboratory (Table 1), and radiological improvement following culture-guided antibiotic therapy, including regression of pulmonary nodules and cervical lymphadenopathy, healing of the discharging sinus, resolution of hepatosplenomegaly and systemic symptoms, and normalization of inflammatory markers. A repeat chest X-ray obtained at discharge showed significant resolution of the bilateral heterogeneous pulmonary opacities (Figure 5). In the absence of microbiological evidence for tuberculosis and with a favourable response to targeted antimicrobial therapy, the overall clinicoradiological profile was considered more consistent with probable systemic involvement associated with *Sphingomonas paucimobilis* infection in an immunocompromised host. The patient was discharged in stable condition after two weeks of hospitalization.

**Figure 5:**
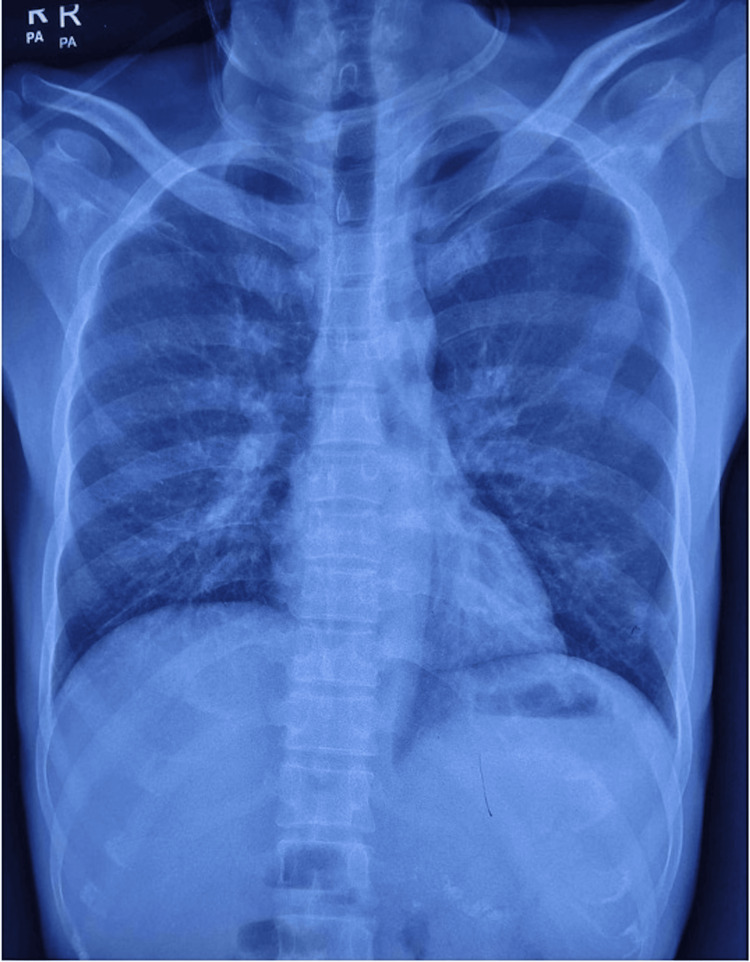
X-ray chest on discharge.

## Discussion

Disseminated *Sphingomonas paucimobilis* infection remains uncommon and can pose a significant diagnostic challenge, especially in tuberculosis-endemic countries such as India. The present case is clinically important because the patient initially fulfilled several classic clinical and radiological features suggestive of disseminated tuberculosis. Constitutional symptoms, including fever, weight loss, anorexia, necrotic cervical lymphadenopathy with discharging sinus, hepatosplenomegaly, and multiple bilateral pulmonary nodules, strongly favoured tuberculosis in the Indian context, where empirical antitubercular therapy is frequently considered in similar presentations. Necrotic cervical lymphadenopathy with sinus formation is particularly regarded as highly suggestive of tuberculous lymphadenitis in endemic settings. However, despite strong clinicoradiological suspicion, repeated microbiological investigations failed to demonstrate Mycobacterium tuberculosis. Acid-fast bacilli staining and CBNAAT from pus, as well as lung aspirate samples, remained negative. In contrast, repeated pus cultures consistently isolated *Sphingomonas paucimobilis*, supporting its pathogenic role rather than contamination. Nevertheless, we acknowledge that negative CBNAAT and FNAC findings alone cannot completely exclude paucibacillary or disseminated tuberculosis, particularly in endemic regions. Pulmonary and reticuloendothelial involvement in the present case, therefore, remained presumptive and was inferred from clinicoradiological findings and significant therapeutic response to targeted antimicrobial therapy without antitubercular treatment.

Alternative differential diagnoses considered in this patient included disseminated fungal infection, metastatic malignancy, inflammatory granulomatous disease, and septic embolic phenomena. Multiple bilateral pulmonary nodules with surrounding ground-glass opacities and occasional spiculated margins can occur in infectious, inflammatory, or neoplastic conditions [3]. Similarly, hepatosplenomegaly and necrotic lymphadenopathy are nonspecific findings with broad differentials. However, fungal microscopy and microbiological investigations were negative, FNAC from the pulmonary nodule showed no evidence of malignancy, and significant regression of pulmonary lesions, lymphadenopathy, hepatosplenomegaly, and systemic symptoms following targeted antibiotic therapy favored an infective etiology associated with *Sphingomonas paucimobilis*.

Another important aspect of this case was the presence of newly detected uncontrolled diabetes mellitus associated with chronic calcific pancreatitis. The diagnosis of type 3c diabetes mellitus was based on the presence of chronic calcific pancreatitis with intraductal and intraparenchymal pancreatic calcifications on imaging, newly detected uncontrolled hyperglycemia, absence of clinical features suggestive of type 1 diabetes mellitus, and the overall clinical context consistent with pancreatogenic diabetes, as described in proposed diagnostic criteria for pancreatogenic diabetes [4]. Diabetes mellitus impairs neutrophil chemotaxis, phagocytosis, and cell-mediated immunity, thereby predisposing patients to opportunistic and atypical infections. While tuberculosis remains one of the most common infectious differential diagnoses in diabetic patients in India [5], clinicians should also consider rare opportunistic pathogens when microbiological findings are inconsistent with tuberculosis.

Recent literature has reported diverse clinical presentations and variable antimicrobial susceptibility patterns in *Sphingomonas paucimobilis* infections [2,6]. Another important finding in this case was the unusual antimicrobial susceptibility profile. The isolate in the present case demonstrated sensitivity to piperacillin-tazobactam and ceftazidime but resistance to carbapenems, fluoroquinolones, and aminoglycosides. The mechanism underlying carbapenem resistance in the present isolate remains unknown, as molecular characterization was not performed. Previous studies have shown heterogeneous susceptibility patterns among *Sphingomonas paucimobilis* isolates [6,7], emphasizing the need for culture-guided therapy rather than empirical escalation to broad-spectrum antibiotics.

Significant clinical, laboratory, and radiological improvement following susceptibility-based antibiotic treatment, including regression of pulmonary nodules and lymphadenopathy with healing of the cervical sinus, further supported the etiological significance of the organism in the present case.

## Conclusions

In tuberculosis-endemic regions, the combination of necrotic lymphadenopathy with sinus formation and pulmonary nodules in an immunocompromised host requires careful diagnostic evaluation. While tuberculosis remains a common diagnostic consideration, culture-guided diagnosis and close clinicoradiological correlation are essential to establish the underlying etiology and avoid unnecessary empirical antitubercular therapy. This case highlights that uncommon opportunistic pathogens such as *Sphingomonas paucimobilis* may occasionally mimic tuberculosis and should be considered when conventional investigations are inconclusive.
